# Distance to Health Centers and Effectiveness of Azithromycin Mass Administration for Children in Niger: A Secondary Analysis of the MORDOR Cluster Randomized Trial

**DOI:** 10.1001/jamanetworkopen.2023.46840

**Published:** 2023-12-15

**Authors:** Dennis L. Chao, Ahmed M. Arzika, Amza Abdou, Ramatou Maliki, Alio Karamba, Nasser Galo, Diallo Beidi, Nasser Harouna, Moustapha Abarchi, Elisabeth Root, Anu Mishra, Elodie Lebas, Benjamin F. Arnold, Catherine E. Oldenburg, Jeremy D. Keenan, Thomas M. Lietman, Kieran S. O’Brien

**Affiliations:** 1 Bill & Melinda Gates Foundation, Seattle, Washington; 2 Centre de Recherche et Interventions en Santé Publique, Birni N’Gaoure, Niger; 3 Programme Nationale de Santé Oculaire, Niamey, Niger; 4 Francis I. Proctor Foundation, University of California, San Francisco; 5 Department of Ophthalmology, University of California, San Francisco; 6 Department of Epidemiology and Biostatistics, University of California, San Francisco; 7 Institute for Global Health Sciences, University of California, San Francisco

## Abstract

**Importance:**

The MORDOR (Macrolides Oraux pour Réduire les Décès avec un Oeil sur la Résistance) trial demonstrated that mass azithromycin administration reduced mortality by 18% among children aged 1 to 59 months in Niger. The identification of high-risk subgroups to target with this intervention could reduce the risk of antimicrobial resistance.

**Objective:**

To evaluate whether distance to the nearest primary health center modifies the effect of azithromycin administration to children aged 1 to 59 months on child mortality.

**Design, Setting, and Participants:**

The MORDOR cluster randomized trial was conducted from December 1, 2014, to July 31, 2017; this post hoc secondary analysis was conducted in 2023 among 594 clusters (communities or *grappes*) in the Boboye and Loga departments in Niger. All children aged 1 to 59 months in eligible communities were evaluated.

**Interventions:**

Biannual (twice-yearly) administration of a single dose of oral azithromycin or matching placebo over 2 years.

**Main Outcomes and Measures:**

A population-based census was used to monitor mortality and person-time at risk (trial primary outcome). Community distance to a primary health center was calculated as kilometers between the center of each community and the nearest health center. Negative binomial regression was used to evaluate the interaction between distance and the effect of azithromycin on the incidence of all-cause mortality among children aged 1 to 59 months.

**Results:**

Between December 1, 2014, and July 31, 2017, a total of 594 communities were enrolled, with 76 092 children (mean [SD] age, 31 [2] months; 39 022 [51.3%] male) included at baseline, for a mean (SD) of 128 (91) children per community. Median (IQR) distance to the nearest primary health center was 5.0 (3.2-7.1) km. Over 2 years, 145 693 person-years at risk were monitored and 3615 deaths were recorded. Overall, mortality rates were 27.5 deaths (95% CI, 26.2-28.7 deaths) per 1000 person-years at risk in the placebo arm and 22.5 deaths (95% CI, 21.4-23.5 deaths) per 1000 person-years at risk in the azithromycin arm. For each kilometer increase in distance in the placebo arm, mortality increased by 5% (adjusted incidence rate ratio, 1.05; 95% CI, 1.03-1.07; *P* < .001). The effect of azithromycin on mortality varied significantly by distance (interaction *P* = .02). Mortality reduction with azithromycin compared with placebo was 0% at 0 km from the health center (95% CI, −19% to 17%), 4% at 1 km (95% CI, −12% to 17%), 16% at 5 km (95% CI, 7% to 23%), and 28% at 10 km (95% CI, 17% to 38%).

**Conclusions and Relevance:**

In this secondary analysis of a cluster randomized trial of mass azithromycin administration for child mortality, children younger than 5 years who lived farthest from health facilities appeared to benefit the most from azithromycin administration. These findings may help guide the allocation of resources to ensure that those with the least access to existing health resources are prioritized in program implementation.

**Trial Registration:**

ClinicalTrials.gov Identifier: NCT02047981

## Key Points

**Question:**

Does the effectiveness of mass administration of azithromycin to children aged 1 to 59 months vary by distance to the nearest primary health center?

**Findings:**

In this secondary analysis of a cluster randomized trial of 594 communities in Niger, the effectiveness of azithromycin mass administration to reduce child mortality varied significantly by distance to the nearest primary health center, with reductions of 0% at 0 km from the health center, 4% at 1 km, 16% at 5 km, and 28% at 10 km.

**Meaning:**

Children in communities farthest from primary health facilities likely benefit the most from mass azithromycin administration to reduce mortality.

## Teaser

This secondary analysis of a cluster randomized trial examines the association among distance to primary health centers, child mortality, and the effectiveness of azithromycin mass drug administration at the community level.

## Introduction

Mortality in children younger than 5 years remains among the highest in the world in West and Central Africa.^1^ Niger has a particularly high mortality rate in those younger than 5 years, with current estimates ranging from 80 to 115 deaths per 1000 live births nationally.^1,2,3^ These estimates are 3 to nearly 5 times the Sustainable Development Goals target of 25 deaths per 1000 live births by 2030.^4^ Reaching such targets will require substantial acceleration of mortality reductions.^5^

Prior work suggests that mortality in those younger than 5 years is associated with distance to health care facilities. Pooled analyses have demonstrated that mortality in infants and children in low- and middle-income settings increases as distance from health care facilities increases, even among those living within 2 km from a facility.^6,7,8^ Several studies in West Africa of caregiver-reported care-seeking behavior similarly found that health care use significantly decreases as distance increases.^9,10,11^ In Niger, cost, distance, and lack of transportation have been reported as major barriers to accessing health care among parents of children who recently died.^11^

Azithromycin mass administration to children younger than 5 years has been found to reduce child mortality in sub-Saharan African settings.^12,13^ The mass drug administration (MDA) approach reduces barriers to access to care by equitably distributing to all children directly in communities, requiring no cost or transportation by caregivers. However, azithromycin MDA has also been found to increase macrolide resistance, leading to caution about widespread implementation of this intervention.^14,15,16^ Conditional guidelines on azithromycin MDA for child survival were released by the World Health Organization in 2020, suggesting that only the highest mortality areas and subpopulations be targeted.^17^ By restricting eligibility to settings with mortality in those younger than 5 years of greater than 80 deaths per 1000 live births or infant mortality of greater than 60 deaths per 1000 live births and limiting distributions to children aged 1 to 11 months, the guidelines aim to include groups at the highest risk of mortality while reducing the risk of antimicrobial resistance.^17^

Using data from the Niger site of the MORDOR (Macrolides Oraux pour Réduire les Décès avec un Oeil sur la Résistance) cluster randomized trial (list of investigators in eAppendix in Supplement 1), we studied the associations between distance to primary health centers, child mortality, and the effectiveness of azithromycin MDA at the community level. The presence of a differential effect of azithromycin MDA on mortality by distance to a primary health center could influence guidelines on implementation of this intervention, including whether to target specific populations to further optimize use of resources and limit the emergence of antimicrobial resistance.

## Methods

This post hoc secondary analysis of the MORDOR trial aimed to examine the association between distance to a primary health center and mortality and to determine the presence of a differential effect of azithromycin MDA on child mortality by distance to a primary health center. This analysis used data from the MORDOR Niger site as well as additional sources to capture relevant spatial information and locations of health facilities in the study area. Verbal consent was obtained from community leaders before study activities commenced and from households before participation in census and intervention activities. For the Niger site, the MORDOR trial was approved by the Niger Ministry of Health and the University of California, San Francisco Human Research Protection Program. Trial oversight was provided by a data and safety monitoring committee. The trial protocol is available in Supplement 1. The current analysis was conducted in 2023 and follows the Consolidated Standards of Reporting Trials (CONSORT) reporting guideline.^18^

### MORDOR Trial: Design, Participants, Interventions, and Data Collection

The original MORDOR trial was conducted from December 1, 2014, to July 31, 2017, in Niger, Malawi, and Tanzania.^12^ Full protocol details have been reported previously.^12^ The current study includes the Niger site because it was the only site with mortality rates that met the World Health Organization criteria for this intervention. In Niger, MORDOR included all children aged 1 to 59 months from 594 *grappes* from the Boboye and Loga departments that had 200 to 2000 inhabitants at the most recent census. *Grappes* are small administrative units in Niger equivalent to a community and hereafter referred to as communities. Communities were randomized in a 1:1 allocation to biannual (twice-yearly) oral azithromycin or matching placebo and were visited by study teams to distribute treatment and monitor vital status approximately every 6 months for 2 years. Participants, investigators, and census workers were masked to allocation. At each census, the eligible population was enumerated, and basic demographic information was collected from household members. At each follow-up census, vital status of children aged 1 to 59 months was recorded as alive, died, moved, or unknown. For the primary mortality outcome, children were counted as deceased if they were present and alive on one census and recorded as died on the subsequent census. Person-time at risk was calculated as the number of days between consecutive censuses, with children who died, moved, or had an unknown status contributing half of that intercensus interval. Global Positioning System coordinates were also recorded for each household. The location of each community was taken to be the median latitude and longitude of all households in the community across census visits. This analysis included data on treatment arm, child vital status, person-time at risk, child age, and community location from the Niger site of the MORDOR trial.

### Additional Data Sources

Locations of roads and rivers were obtained from OpenStreetMap (OpenStreetMap Foundation). Primary health center locations were available from Carte Sanitaire du Niger (Ministère de la Santé Publique de la Population et des Affaires Sociales, Niamey, Niger). This analysis included location data on Centre de Santé Integré facilities, which are primary health centers that serve catchment areas of approximately 5 to 10 communities and are equipped to provide basic inpatient and outpatient care.^19^ Each community was linked with its nearest primary health center as determined by Euclidean distance. Department boundaries were obtained from the GADM database of Global Administrative Areas, version 4.1 (GADM).

### Statistical Analysis

This secondary analysis was conducted in 2023. The sample size for this analysis was fixed by enrollment in the MORDOR Niger trial, which was powered to detect a community-level effect of azithromycin MDA on mortality among children aged 1 to 59 months over 2 years. Details of sample size calculations have been reported previously.^12^

Descriptive summaries included community-level baseline characteristics by arm using numbers (percentages) for categorical variables and means (SDs) or medians (IQRs) for continuous variables. Crude mortality rates were calculated as the count of deaths per community divided by the community-level person-time at risk, and 95% CIs were computed assuming a Poisson process. Crude mortality rates were examined by distance to a primary health center, which was categorized in 1-km increments up to 10 km.

Negative binomial regression was used to examine the association of azithromycin MDA with mortality by distance to a primary health center at the community level. Models included the number of deaths per community as the outcome, covariates for treatment arm, mean child age per community at baseline, distance to a primary health center, and the interaction between treatment arm and distance to examine multiplicative effect modification. Community-level person-time at risk was included as an offset. Mean community age was included as a covariate because age is a known risk factor for mortality, with younger ages experiencing higher mortality. Community-level clustering was taken into account by the dispersion parameter in the model. To aid interpretation of the interaction, the association of azithromycin MDA with mortality was estimated at specific distances from health centers (1 km, 5 km, and 10 km). The 5-km increments were chosen because they align with thresholds at which the Niger health system distributes different community services. A sensitivity analysis was conducted using Poisson regression models in a similar format with robust SEs to account for clustering. The Johnson-Neyman procedure was used to determine regions of significance assuming an α of .05.^20^ In addition, the empirical mortality rate difference, number needed to treat to avert 1 death, and number of deaths averted were estimated by the same categories of distance to the primary health center. All analyses were conducted in R, version 4.2.2 (R Project for Statistical Computing) and used an α of .05 to determine statistical significance.

## Results

**Figure 1.**
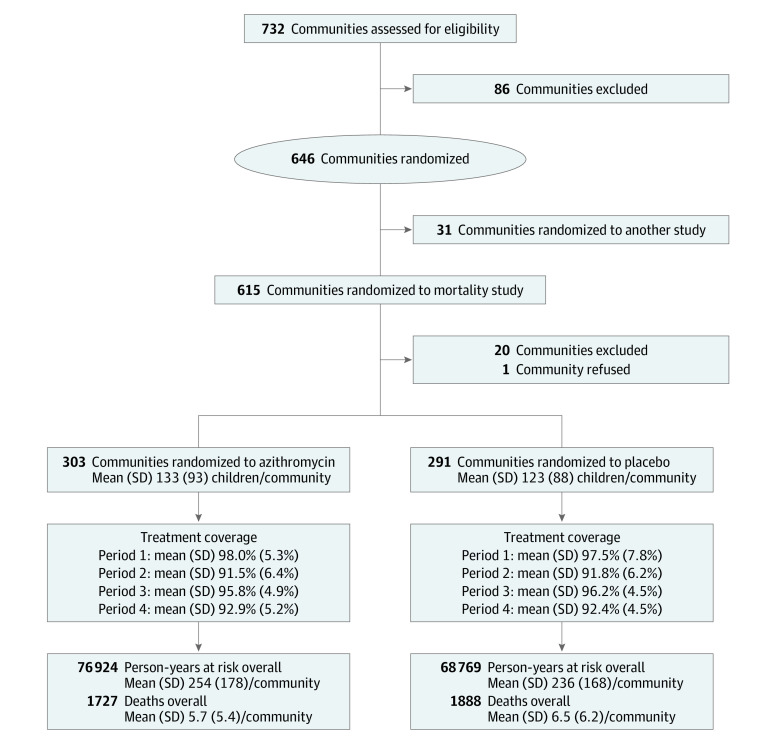
Participant Flow Diagram

**Figure 2.**
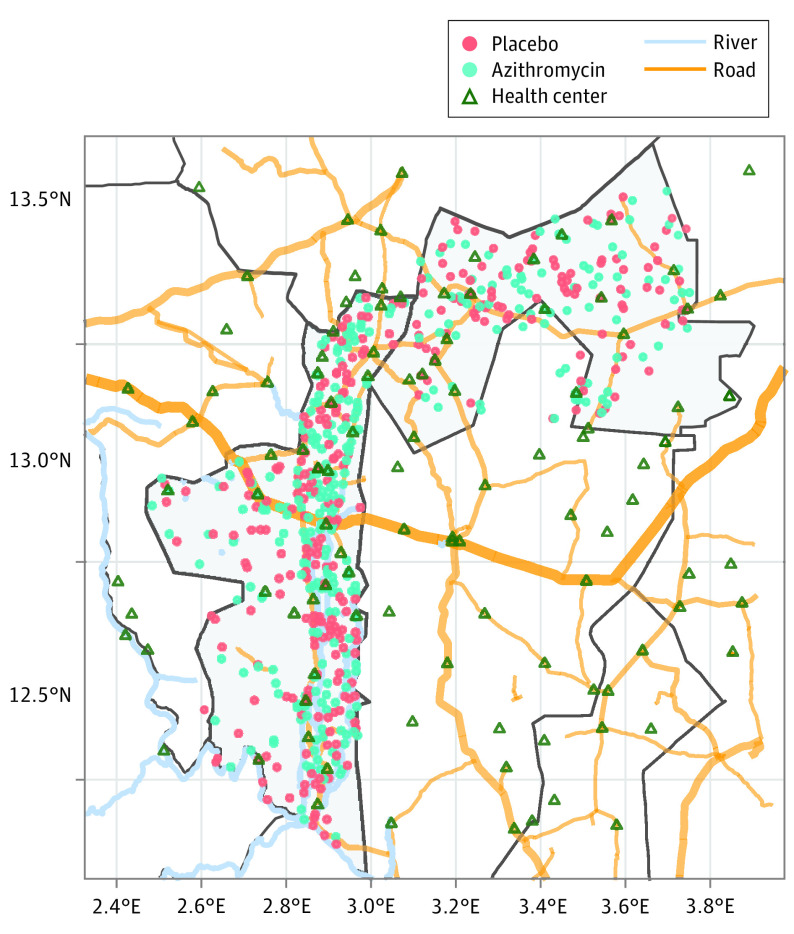
Map of Communities Included in the MORDOR Niger Trial From 2014 to 2017 MORDOR indicates Macrolides Oraux pour Réduire les Décès avec un Oeil sur la Résistance.

Between December 1, 2014, and July 31, 2017, a total of 594 communities were enrolled, with 76 092 children (mean [SD] age, 31 [2] months; 39 022 [51.3%] male and 37 070 [48.7%] female) included at baseline for a mean (SD) of 128 (91) children per community (Figure 1). Baseline characteristics of communities were similar by arm (Table 1). Assuming that communities use the nearest primary health center, 51 primary health centers were identified as serving the 594 included communities. Overall, the median (IQR) distance of communities to the nearest primary health center was 5.0 (3.2-7.1) km (eFigure 1 in Supplement 2). A map of included communities and primary health centers is shown in Figure 2.

**Table 1.** Baseline Characteristics of Communities Included in the MORDOR Niger Trial by Treatment Arm

Characteristic	Placebo	Azithromycin
Communities, No.	291	303
Children aged 1-59 mo, No.	35 747	40 345
Primary health centers, No.	49	48
Children aged 1-59 mo per community, mean (SD), No.	123 (88)	133 (93)
Age at the community level, mean (SD), mo	31.1 (2.1)	30.9 (2.3)
Distance from primary health center, median (IQR), km	5.3 (3.3-7.6)	4.8 (3.2-6.8)

Abbreviation: MORDOR, Macrolides Oraux pour Réduire les Décès avec un Oeil sur la Résistance.

**Figure 3.**
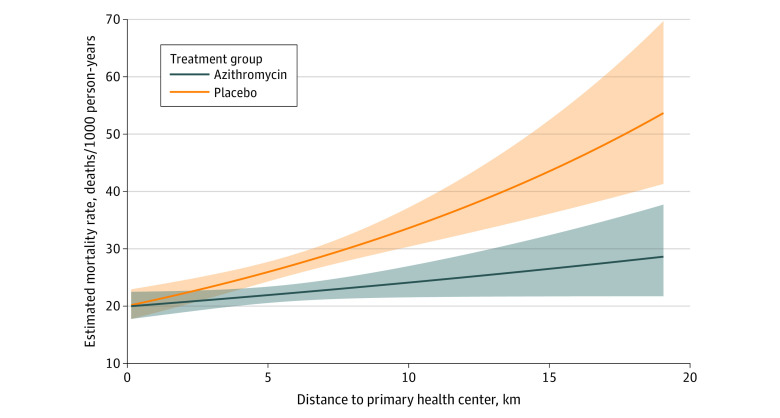
Association Between Distance to a Primary Health Center and Mortality Incidence Rate Values from a negative binomial regression model estimating the mortality incidence rate by treatment arm and distance to a primary health center, controlling for community mean age at baseline. Shaded areas indicate 95% CIs.

The analysis included 3615 deaths and 145 693 person-years at risk (Figure 1). As previously reported, overall mortality rates were 27.5 deaths per 1000 person-years at risk (95% CI, 26.2-28.7) in the placebo arm and 22.5 deaths per 1000 person-years at risk (95% CI, 21.4-23.5) in the azithromycin arm.^12^ For each kilometer increase in community distance to the nearest primary health center in the placebo arm, mortality increased by 5% (adjusted incidence rate ratio, 1.05; 95% CI, 1.03-1.07; *P* < .001) (Table 2 and Figure 3). Interaction between the association of azithromycin MDA and distance with mortality was statistically significant on the multiplicative scale (interaction *P* = .02) (Table 2). Mortality reduction with azithromycin compared with placebo was 0% at 0 km from the health center (95% CI, −19% to 17%), 4% at 1 km (95% CI, −12% to 17%), 16% at 5 km (95% CI, 7%-23%), and 28% at 10 km (95% CI, 17%-38%). Sensitivity analyses produced similar results (eTable 1 in Supplement 2). At distances of 3.25 km and above, the association of azithromycin MDA compared with placebo with mortality was statistically significant. Figure 3 displays the estimated mortality rates by treatment arm and distance. Observed mortality rates by treatment arm and distance category are given in eTable 2 and eFigure 2 in Supplement 2.

**Table 2.** Evaluation of Heterogeneity of Effect of Azithromycin vs Placebo Distribution on Child Mortality by Distance^a^

Distance to primary health center, km	Adjusted incidence rate ratio (95% CI)^b^		
	Placebo	Azithromycin	Azithromycin vs placebo within strata of distance
0	1 [Reference]	1.00 (0.83-1.19)	1.00 (0.83-1.19)
1	1.05 (1.03-1.07)	1.01 (0.86-1.07)	0.96 (0.83-1.12)
5	1.29 (1.18-1.43)	1.09 (0.95-1.43)	0.84 (0.77-0.93)
10	1.68 (1.38-2.03)	1.20 (1.01-2.03)	0.72 (0.62-0.83)

^a^ To aid interpretation of the interaction, outcomes were estimated at specific distances from health centers that were chosen to align with thresholds at which the Niger health system distributes different community services.

^b^ Adjusted incidence rate ratios were estimated using negative binomial regression and adjusted for community mean age. For the measure of effect modification on a multiplicative scale, the ratio of adjusted incidence rate ratios was 0.97 (95% CI, 0.95-0.99; *P* = .02).

Similarly, the incidence rate difference in mortality increased as distance increased (eTable 2 in Supplement 2). The number needed to treat to avert 1 death therefore decreased as distance increased, with numbers needed to treat of 223 (95% CI, 142-516) in the 1- to less than 5-km range, 167 (95% CI, 117-292) in the 5- to less than 10-km range, and 72 (95% CI, 48-144) in the 10-km or greater range from the nearest primary health center.

## Discussion

In this secondary analysis of the MORDOR Niger cluster randomized trial, we evaluated associations among distance to a primary health center, child mortality, and the effectiveness of azithromycin MDA to reduce child mortality. Mortality among children aged 1 to 59 months increased sharply with distance from a primary health center, with a 5% increase in mortality found for every 1-km increase in distance. The effectiveness of azithromycin MDA to reduce child mortality also increased with distance. No association was demonstrated among those living closest to the primary health care centers, and increasing reductions in mortality were found as distance increased, from a 16% reduction at a distance of 5 km to a 28% reduction at 10 km.

A similar association between distance to health care and mortality among children has been identified in several studies in low- and middle-income settings.^6,7,8,21^ In these analyses, distance may be a proxy for access to and/or utilization of health centers and their services, including access to antibiotics and other life-saving care. In fact, other studies demonstrate that health center utilization for children younger than 5 years decreases as distance increases, even for the leading causes of mortality, such as pneumonia, diarrhea, and malaria.^9,10,11,22,23,24^ One such study in Burkina Faso found that the majority of completed child health care visits resulted in an antibiotic prescription.^22^ An understanding of the impact of distance to health resources led the Niger Ministry of Public Health to scale up community-based health care services in the early 2000s, resulting in a substantial increase in geographic access to primary health centers between 2000 and 2013.^25^ However, even with this expansion, 59% of the population remained beyond a 60-minute walking distance to the nearest primary health center.^25^

Azithromycin is a broad-spectrum antibiotic, and its effectiveness in reducing child mortality in this setting likely has numerous mechanisms. Azithromycin MDA has been associated with reductions in morbidity and mortality related to diarrheal disease, respiratory infections, and malaria.^26,27,28,29^ Given the possible mechanisms, several potential explanations exist for the association between azithromycin effectiveness and distance. Children living close to primary health centers may be more likely to be vaccinated or receive life-saving care when needed, in addition to receiving multiple doses of antibiotics each year. With the increased access to antibiotics, it is also possible that communities closest to the primary health centers have higher existing antibiotic resistance, reducing the effectiveness of azithromycin MDA at the shortest distances. Other studies have identified a similar association between distance to a health facility and effectiveness of pneumococcal conjugate vaccination, finding no difference in vaccine effectiveness for those close to health care and an increasing effectiveness as distance increased, similarly suggesting that groups with the least access to care might be most in need of intervention.^30,31^

Concern about the potential for azithromycin MDA to increase antimicrobial resistance has led to attempts to identify high-risk subgroups for targeting to limit antibiotic distributions.^17^ Several studies have examined effect heterogeneity by baseline mortality, age group, anthropometric indicators, and other indicators of spatial heterogeneity.^12,32,33,34^ Although not definitive, these studies suggested greater effectiveness in the higher-risk subgroups, including younger age, settings with higher background mortality, and those with lower weight. As a result, the World Health Organization restricted eligibility for this intervention to children aged 1 to 11 months in the highest mortality settings.^17^ Regardless of whether used to justify further targeting, these results support the use of resources to reach the farthest communities to maximize the effectiveness and equity of this intervention.

### Strengths and Limitations

Strengths of this study include the cluster randomized trial design, which involved a randomized and masked intervention, standardized and rigorous data collection procedures, and a low likelihood of misclassification of exposure, outcome, or other variables. In addition, the large sample size enabled examination of effect modification on mortality, which is a relatively rare event even in high-mortality settings. This study also included a population-based sample, which ensures generalizability to similar settings within the range of eligible community size. However, the study also has some limitations. MORDOR did not include communities smaller than 200 or larger than 2000 people; thus, results may not generalize beyond this range. We also used Euclidean distance to the nearest primary health center in this study as a proxy for access to a health center, whereas other methods, such as friction surfaces, may reflect travel times more accurately.^35^ This choice may not have impacted our results greatly because in this setting in Niger, many travel by walking on small roads. This analysis did not consider seasonality, which may also play an important role in travel time and health facility access. In addition, if the available data on health care facility location were incomplete or out of date, there may be some location misclassification that could impact results, although we would not expect this to differ by intervention arm. Finally, because this effect heterogeneity analysis aimed to identify subgroups that could be targeted, only confounding of the primary exposure-outcome association must be considered, which we would not expect given the randomized intervention.^36^ Nevertheless, we did adjust for age given the strong association between age and mortality and found similar results in unadjusted and adjusted models.

## Conclusions

In this secondary analysis of a cluster randomized trial, we found that the effectiveness of azithromycin MDA to reduce child mortality increased significantly as distance to the nearest primary health center increased. These findings suggest that resources should be allocated to ensure that those with the least access to existing health resources are prioritized in program implementation.

## Notes

Supplement 1.
Trial Protocol and Statistical Analysis Plan

Supplement 2. **eAppendix.** MORDOR-Niger Study Group Investigators **eFigure 1.** Number of Children by Distance to Nearest Primary Health Center **eFigure 2.** Observed Child Mortality Rates by Treatment Arm and Distance to the Nearest Primary Health Center **eTable 1.** Unadjusted and Adjusted Results From Negative Binomial and Poisson Regression Models to Estimate the Effect of Treatment and Distance to Primary Health Center on Mortality Rate **eTable 2.** Estimates of Differences in Mortality Incidence Rate Between Azithromycin and Placebo Communities, Number Needed to Treat to Prevent One Death, and Deaths Averted If All Had Received Azithromycin by Distance to Primary Health Center

Supplement 3.
Data Sharing Statement
