# Supplementary material for: Distance to Health Centers and Effectiveness of Azithromycin Mass Administration for Children in Niger: A Secondary Analysis of the MORDOR Cluster Randomized Trial
Source: JAMA Netw Open. 2023 Dec 15;6(12):e2346840. doi: 10.1001/jamanetworkopen.2023.46840 (PMC10724761; doi:10.1001/jamanetworkopen.2023.46840)
Supplement: Supplement 2. — eAppendix. MORDOR-Niger Study Group Investigators eFigure 1. Number of Children by Distance to Nearest Primary Health Center eFigure 2. Observed Child Mortality Rates by Treatment Arm and Distance to the Nearest Primary Health Center eTable 1. Unadjusted and Adjusted Results From Negative Binomial and Poisson Regression Models to Estimate the Effect of Treatment and Distance to Primary Health Center on Mortality Rate eTable 2. Estimates of Differences in Mortality Incidence Rate Between Azithromycin and Placebo Communities, Number Needed to Treat to Prevent One Death, and Deaths Averted If All Had Received Azithromycin by Distance to Primary Health Center [file jamanetwopen-e2346840-s002.pdf]

Supplementary Online Content

Chao DL, Arzika AM, Amza A, et al. Distance to health centers and effectiveness of azithromycin mass administration for children in Niger: a secondary analysis of the MORDOR cluster randomized trial. *JAMA Netw Open*. 2023;6(12):e2346840. doi:10.1001/jamanetworkopen.2023.46840

eAppendix. MORDOR-Niger Study Group Investigators

eFigure 1. Number of Children by Distance to Nearest Primary Health Center

eFigure 2. Observed Child Mortality Rates by Treatment Arm and Distance to the Nearest Primary Health Center

eTable 1. Unadjusted and Adjusted Results From Negative Binomial and Poisson Regression Models to Estimate the Effect of Treatment and Distance to Primary Health Center on Mortality Rate

eTable 2. Estimates of Differences in Mortality Incidence Rate Between Azithromycin and Placebo Communities, Number Needed to Treat to Prevent One Death, and Deaths Averted If All Had Received Azithromycin by Distance to Primary Health Center

This supplementary material has been provided by the authors to give readers additional information about their work.

© 2023 Chao DL et al. *JAMA Network Open.*

MORDOR-Niger Study Group Investigators

University of California, San Francisco, San Francisco, CA, USA
Catherine Cook, Sun Y Cotter, Thuy Doan, Dionna M Fry, Jeremy D Keenan, Elodie Lebas, Thomas M Lietman, Ying Lin, Kieran S O'Brien, Catherine E Oldenburg, Travis C Porco, Kathryn J Ray, Philip J Rosenthal, George W Rutherford, Benjamin Vanderschelden, Nicole E Varnado, Lina Zhong, Zhaoxia Zhou

The Carter Center, Atlanta, GA, USA
E Kelly Callahan, Aisha E Stewart

The Carter Center Niger, Niamey, Niger
Ahmed M Arzika, Sanoussi Elh Adamou, Nana Fatima Galo, Fatima Ibrahim, Salissou Kane, Mariama Kiemago, Ramatou Maliki

Programme National de Santé Oculaire, Niamey, Niger
Amza Abdou, Boubacar Kadri, Nassirou Beido

London School of Hygiene and Tropical Medicine, London, UK
Robin L Bailey, John Hart

Johns Hopkins University, Baltimore, MD, USA
Jerusha Weaver, Sheila K West

International Trachoma Initiative, Decatur, GA, USA
Paul M Emerson

Steering Committee. The steering committee for the trial consisted of Robin L Bailey, Jeremy D Keenan, Thomas M Lietman, Travis C Porco, and Sheila K West.

Sponsor program officers. The program officers from the trial's sponsor included Rasa Izadnegahdar, Julie Jacobson, Thomas Kanyok, and Erin Shutes (Bill & Melinda Gates Foundation, Seattle, WA, USA).

Data and Safety Monitoring Committee (DSMC). The trial's DSMC consisted of Judd L Walson (University of Washington, Seattle, WA, USA), Allen W Hightower (Centers for Disease Control and Prevention, Atlanta, GA, USA), Emily E Anderson (Loyola University, Chicago, IL, USA), Wondu Alemayehu (Fred Hollows Foundation, Addis Ababa, Ethiopia), and Latha Rajan (Tulane University, New Orleans, LA, USA)

© 2023 Chao DL et al. *JAMA Network Open.*

eFigure 1. Number of children by distance to nearest primary health center.

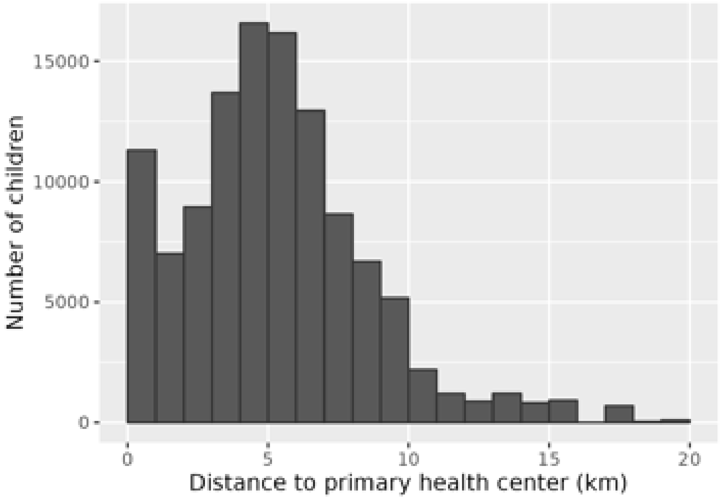

© 2023 Chao DL et al. *JAMA Network Open.*

eFigure 2. Observed child mortality rates by treatment arm and distance to the nearest primary health center. Distance was divided into 1 km bins, up to 10 km, beyond which all participants are combined. Mortality is the number of deaths divided by person-time at risk, and 95% confidence intervals were computed assuming a Poisson process.

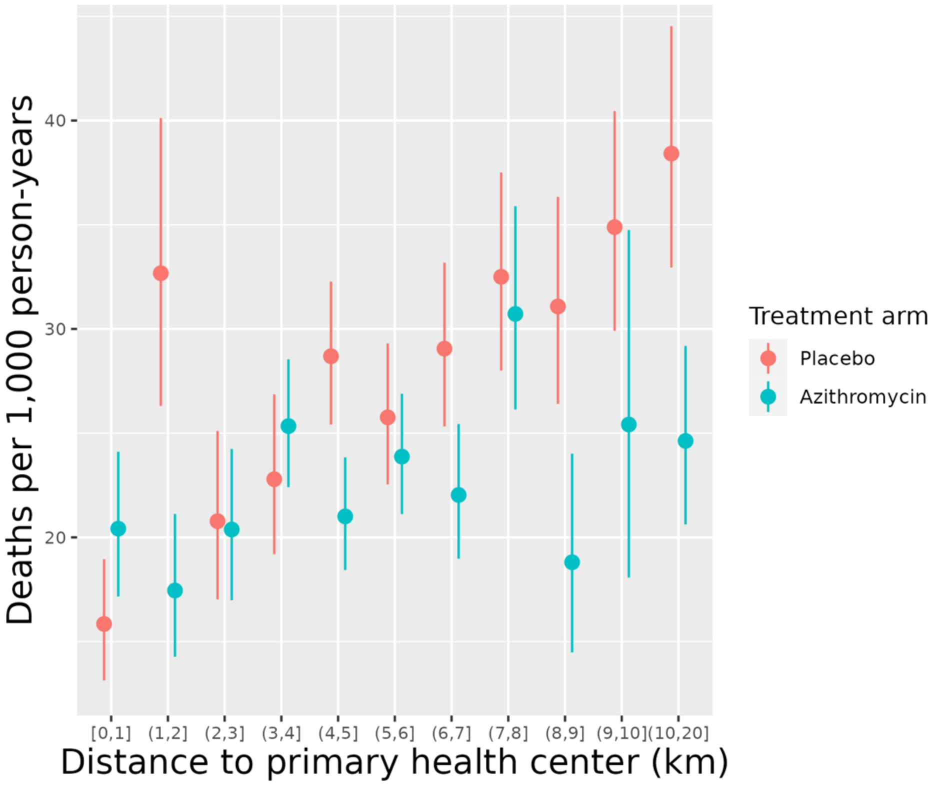

© 2023 Chao DL et al. *JAMA Network Open.*

eTable 1. Unadjusted and adjusted results from negative binomial and Poisson regression models to estimate the effect of treatment and distance to primary health center on mortality rate. Negative binomial regression was used in the primary analyses and Poisson regression with robust standard errors was used in sensitivity analyses.

**Model and Variable**	**Unadjusted**			**Adjusted**		
	**IRR**	**95% CI**	***P*-value**	**IRR**	**95% CI**	***P*-value**
Negative binomial model						
Treatment arm	1.03	0.85 to 1.23	0.78	1.00	0.83 to 1.19	0.96
Distance to primary health center	1.06	1.04 to 1.08	< 0.001	1.05	1.03 to 1.07	< 0.001
Average age at baseline	NA	NA	NA	0.95	0.93 to 0.97	< 0.001
Treatment arm and distance interaction	0.97	0.94 to 0.99	0.01	0.97	0.94 to 0.99	0.02
Poisson model						
Treatment arm	1.03	0.85 to 1.25	0.77	0.99	0.82 to 1.20	0.94
Distance to primary health center	1.06	1.03 to 1.08	< 0.001	1.05	1.03 to 1.07	< 0.001
Average age at baseline	NA	NA	NA	0.95	0.93 to 0.97	< 0.001
Treatment arm and distance interaction	0.96	0.94 to 0.99	0.01	0.97	0.94 to 0.99	0.02

CI, confidence interval; IRR, incidence rate ratio

© 2023 Chao DL et al. *JAMA Network Open.*

eTable 2. Estimates of differences in mortality incidence rate between azithromycin and placebo communities, number needed to treat to prevent one death, and deaths averted if all had received azithromycin by distance to primary health center.

Distance category (km)	Azithromycin			Placebo			Incidence Rate Difference (95% CI)	Number needed to treat to avert one death[1] (95% CI)	Number of deaths averted (95% CI)
	Deaths (n)	Person-years at risk	Deaths per 1,000 person years (95% CI)	Deaths (n)	Person-years at risk	Deaths per 1,000 person years (95% CI)			
0 to < 1	138	6,851	20.1 (17.0 to 23.8)	120	7,634	15.7 (13.1 to 18.8)	4.4 (0 to 8.8)	NA[2]	NA[2]
1 to < 5	739	34,568	21.4 (19.9 to 23.0)	621	24,007	25.9 (23.9 to 28.0)	-4.5 (-1.9 to -7.0)	223 (142 to 516)	263 (113 to 412)
5 to < 10	718	30,112	23.8 (22.2 to 25.7)	971	32,551	29.8 (28.0 to 31.8)	-6.0 (-3.4 to -8.6)	167 (117 to 292)	375 (215 to 536)
≥ 10	132	5,393	24.5 (20.6 to 29.0)	176	4,578	38.4 (33.2 to 44.6)	-14.0 (-6.9 to -21.0)	72 (48 to 144)	139 (69 to 210)

CI, confidence interval; km, kilometer

[1]Number needed to treat with azithromycin MDA estimated using incidence rates and interpreted as number of children treated per year over 2 years required to avert one death
[2]Not calculated as no effect of azithromycin MDA on mortality incidence compared to placebo was demonstrated for the 0-1 km group

© 2023 Chao DL et al. *JAMA Network Open.*
