# Supplementary material for: Distance to Health Centers and Effectiveness of Azithromycin Mass Administration for Children in Niger: A Secondary Analysis of the MORDOR Cluster Randomized Trial
Source: JAMA Netw Open. 2023 Dec 15;6(12):e2346840. doi: 10.1001/jamanetworkopen.2023.46840 (PMC10724761; doi:10.1001/jamanetworkopen.2023.46840)
Supplement: Supplement 3. — Data Sharing Statement [file jamanetwopen-e2346840-s003.pdf]

Data Sharing Statement

Chao. Distance to Health Centers and Effectiveness of Azithromycin Mass Administration for Children in Niger. *JAMA Netw Open*. Published December 13, 2023.
doi:10.1001/jamanetworkopen.2023.46840

Data
Data available: Yes
Data types: Deidentified participant data
How to access data: The de-identified data used in this analysis and accompanying codebook are publicly available at Open Science Framework (https://osf.io/m6eh8/).
When available: beginning date: 06-01-2023

Supporting Documents
Document types: None

Additional Information
Who can access the data: The data are publicly available.
Types of analyses: For any purpose
Mechanisms of data availability: The data are publicly available.
